#  Component Analysis of Iranian Crack; A Newly Abused Narcotic Substance in Iran

**Published:** 2014

**Authors:** Ali Farhoudian, Mandana Sadeghi, Hamid Reza Khoddami Vishteh, Babak Moazen, Monir Fekri, Afarin Rahimi Movaghar

**Affiliations:** a *Iranian Research Center for Substance Abuse and Dependence, University of Social Welfare and Rehabilitation Science, Tehran, Iran. *; b *IRSA Center for Psychology and Addiction Study, Tehran, Iran. *; c *Non-Communicable Diseases Research Center, Endocrinology and Metabolism Population Sciences Institute, Tehran University of Medical Sciences, Tehran, Iran, *; d *Endocrinology and Metabolism Research Center, Endocrinology and Metabolism Clinical Sciences Institute, Tehran University of Medical Sciences, Tehran, Iran,*; e *Bahar Laboratory of Toxicology, Tehran, Iran.*^*f*^*Iranian Research Center for HIV/AIDS, Tehran University of Medical Sciences, Tehran, Iran.*

**Keywords:** Substance use, Heroin, Crack cocaine, Iranian crack, Crack, TLC, HPLC

## Abstract

Iranian crack is a new form of narcotic substance that has found widespread prevalence in Iran in the past years. Crack only nominally resembles crack cocaine as it is widely different in its clinical signs. Thus the present study aims to quantify the chemical combination of this drug. The samples included 18 specimen of Crack collected from different zones of Tehran, Iran. All specimens were in the form of inodorous cream solid powdery substance. TLC and HPLC methods were used to perform semi-quantitative and quantitative analysis of the components, respectively.

The TLC analysis showed no cocaine compound in the specimens while they all revealed to contain heroin, codeine, morphine and caffeine. All but two specimens contained thebaine. None of the specimens contained amphetamine, benzodiazepines, tricyclic antidepressants, aspirin, barbiturates, tramadol and *buprenorphine*. Acetaminophen was found in four specimens. HPLC revealed heroin to be the foundation substance in all specimens and most of them contained a significant amount of acetylcodeine. The present analysis of the chemical combination of Crack showed that this substance is a heroin-based narcotic which is basically different from the cocaine-based crack used in Western countries. Studies like the present one at different time points, especially when abnormal clinical signs are detected, can reveal the chemical combination of the target substance and contribute to the clinical management of its acute or chronic poisoning.

## Introduction

Crack is the nickname for the freebase form of alkaloid cocaine which is known as a dangerous addictive drug. Through wreaking adverse effect on the central nervous system it results in symptoms such as insomnia, euphoria, alertness and increased energy. Recently, a new form of narcotic has been widely used in Iran with the same name of crack (1), while, according to the addiction treatment practitioners reports that it’s clinical and withdrawal signs are quite different from those of the common crack cocaine (2). Unlike the clinical signs of common crack cocaine, using Iranian crack causes pupil constriction, stress reduction and overt sleepiness. Several hours after use, the patient undergoes symptoms such as obsession and other psychological signs and also physiological signs such as rhinorrhea, *epiphoria*, pupillary dilation and pain which can be restored by using methadone or *buprenorphine.* Unlike cocaine, which is a pleasant additive to use with other narcotic substances, the desire to use Crack decreases or is gone through treatment with other narcotics? This shows that the chemical combination of Crack is different from that of the common cocaine because its symptoms and side effects are similar to heroin instead of cocaine. So it seems that the similarity between these two substances is in pronunciation not chemical content. 

Although there is no official report on the prevalence of Crack abuse in Iran, it seems that due to its inodorousness, simple and rapid preparation, ease of use and highly addictiveness this drug must be very common in Iran (2). Narenjiha *et al*. report that Crack only comes second to opium in the prevalence of use among truckers and professional drivers (3). Therefore, the widespread use of Crack in Iran has become a serious problem in drug abuse in the Iranian society (2). Since identifying the components of the Iranian crack can help the treatment of the clinical signs of patients, the present study was conducted to analyze the chemical components of this drug. Apart from a previous qualitative study (4) it is notable that this is the only quantitative study on the chemical components of the Iranian crack .

## Experimental

*Materials*

The present study was conducted at Bahar Toxicology Laboratory in Tehran. The samples included 18 specimen of Crack collected from different zones of Tehran, Iran and from addicts who sought treatment in addiction treatment centers. To analyze the chemical combination of samples, 0.05 grams of each powdered sample dissolved in 10 cc of 99% methanol and then extracted by means of 99% chloroform solution. The extracted Crack samples at 23-28 °C were centrifuged at 13,000 rpm for 10 seconds to separate the impurities. The upper phase solution was then scaled through Thin Layer Chromatography (TLC) and High-Performance Liquid Chromatography (HPLC) (5, 6). TLC was performed for the semi-quantitative analysis of the samples for the compounds such as morphine, codeine, thebaine, cannabis, methadone, buprenorphine, amphetamines, cocaine, tramadol, aspirin, diphenoxylate, benzodiazepines, trihexyphenidyl, biperiden, tricyclic antidepressant, caffeine and barbiturates. HPLC was then performed to analyze quantitatively those compounds which were revealed through TLC. The samples were injected into a Japan-made Shimadzu analyzer (SDP-10A-VP) containing methanol, acetonitrile and bisphosphonates at the flow rate of 1 mL/min and a volume of 20 µL and were analyzed with 210-nm UV light. The HPLC column was made of C18 resin. The methanol and chloroform standards and buffers were made by Merk (Germany) or Romil (England). The heroin sample standard was procured from T and H SMITH Ltd., the tramadol sample from Tehran Pharmaceutical Company and remaining standards were procured from Iranian Temad Company. Since there was no standard sample available for acetylcodeine (6-monoacetyle morphine), its concentration was calculated indirectly by division of the heroin (diamorphine) under the curve area to the under the curve area of acetylcodeine and multiplying the result by the heroin concentration. For calibration in HPLC method, 1000 µg form the standard materials was removed and it was dissolved in a suitable solvent (stoke). Then a certain amount of stock removed, and the dilution was prepared in three concentrations between minimum and maximum sensitivity. Each solution was injected three times into the machine. The mean concentration was calculated and the standard curve was set. Certain amount of unknown samples were taken and dissolved in a suitable solvent and it was injected into the machine. The result was compared with the standard curve. Standard curves for stocks and samples were obtained through both the extraction and direct methods which was similar. The data were analyzed by Autochrome v.1, 2000.

## Results

All specimens were solid and would crush into powder if pressed between two fingers. They had creamy color. However, the specimens differed slightly in shades of color. They were all odorless. One specimen, which was obtained from an addicted patient, was partially used and stuck to a safety pin. The used part was coal black and dry. In another specimen, opium could be observed with the naked eye. Table 1 shows the results of different substances in the Crack samples using TLC.

**Table 1:** TLC results of Kearck samples

**Sample No. **	**Morphine**	**Codeine**	**Heroine**	**Thebaine**	**Methadone**	**Cocaine Tramadol**	**Diphenoxylate**	**Trihexyphenidyl**	**Biperiden**	**Amphetamine**	**Benzodiazepines**	**Tricyclic antidepressants**	**Aspirin Caffeine**	**Phenobarbital**	**Acetaminophen**	**Buprenorphine**	**Tramadol**	**Caffeine**
1	+	+	+	+	-	-	-	-	-	-	-	-	-	-	+	-	+	-
2	+	+	+	+	-	-	-	-	-	-	-	-	-	-	+	-	-	-
3	-	+	+	-	-	-	-	-	-	-	-	-	-	-	+	-	-	-
4	+	+	+	+	-	-	-	-	-	-	-	-	-	-	+	-	-	-
5	+	+	+	+	-	-	-	-	-	-	-	-	-	-	+	-	-	-
6	+	+	+	+	-	-	-	-	-	-	-	-	-	-	+	-	-	-
7	+	+	+	+	-	-	-	-	-	-	-	-	-	-	+	-	-	-
8	+	+	+	+	-	-	-	-	-	-	-	-	-	-	+	-	+	-
9	+	+	+	+	-	-	-	-	-	-	-	-	-	-	+	-	-	-
10	+	+	+	+	-	-	-	-	-	-	-	-	-	-	+	-	-	-
11	+	+	+	+	-	-	-	-	-	-	-	-	-	-	+	-	-	-
12	+	+	+	+	-	-	-	-	-	-	-	-	-	-	+	-	-	-
13	+	+	+	+	-	-	-	-	-	-	-	-	-	-	+	-	-	-
14	+	+	+	+	-	-	-	-	-	-	-	-	-	-	+	-	-	-
15	+	+	+	+	-	-	-	-	-	-	-	-	-	-	+	-	+	-
16	+	+	+	-	-	-	-	-	-	-	-	-	-	-	+	-	+	-
17	+	+	+	+	-	-	-	-	-	-	-	-	-	-	+	-	-	-
18	+	+	+	+	-	-	-	-	-	-	-	-	-	-	+	-	-	-

**Figure 1:**
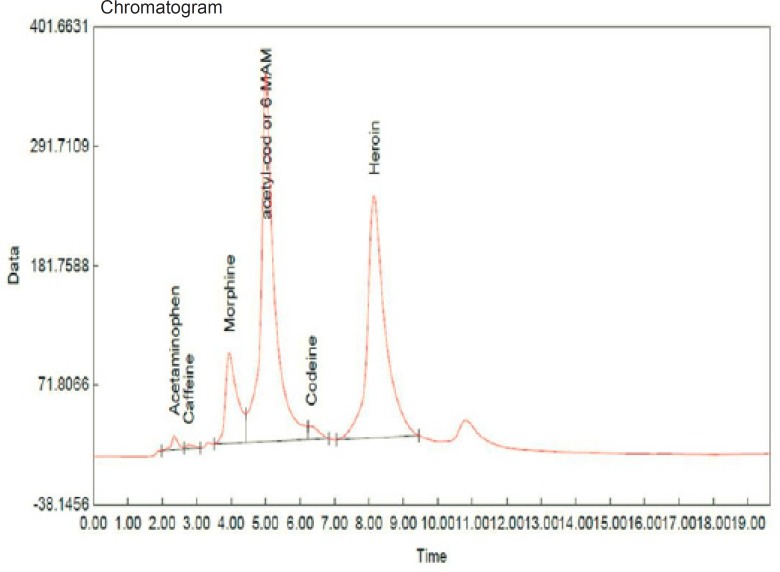
Chromatogram of sample No. 15

Obviously, none of the specimens contained cocaine while they all contained heroin, codeine, morphine and caffeine. All but two specimens contained thebaine. None of the specimens contained amphetamine, benzodiazepines, tricyclic antidepressants, aspirin, barbiturates, tramadol and *buprenorphine*. Acetaminophen was found in four specimens. Figure 1 shows the chromatogram of sample No. 15.

**Figure 2:**
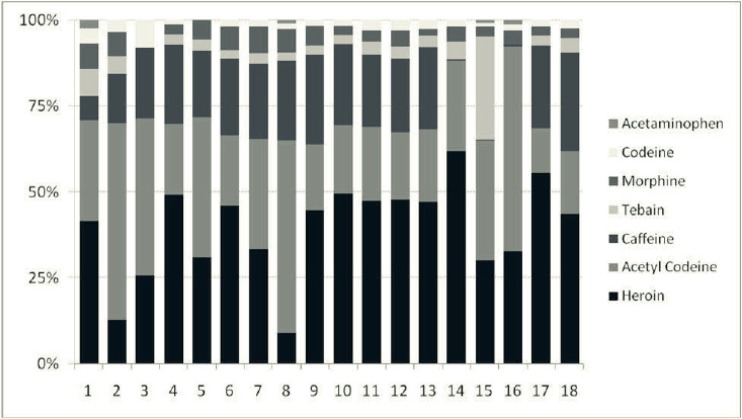
Percentage of chemical components of Crack samples in HPLC

**Figure 3:**
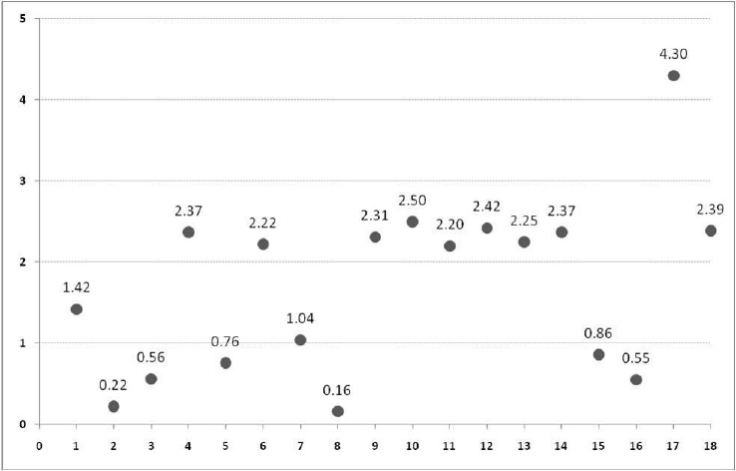
Heroin to acetylcodeine ratio in Crack samples

Table 2 and Figure 2 show the quantitative analysis of the chemical components in the Crack sample using HPLC. As can be observed, all specimens were based on heroin and most showed the substantial combination of acetylcodeine. Figure 3 illustrates the proportion of heroin to acetylcodeine.

**Table 2:** HPLC results (ng/mL) for quantitative concentration of chemical compounds in Crack samples.

**Sample No.**	**Heroin**	**Acetaminophen**	**Caffeine**	**Morphine**	**Codeine**	**Tebain**	**Acetyl-codeine** (6-Mono Acetyl Morphine)*	Dia-morphine/Acetyl codeine under the curve area**
1	256.95	13.84	42.76	46.02	27.75	48.53	180.95	1.42
2	106.83	0	122.03	59.98	28.36	43.16	485.60	0.22
3	94.94	0	76.24	0	29.79	0	169.53	0.56
4	240.58	0	112.89	13.77	5.83	15.28	101.51	2.37
5	120.33	0	74.97	21.72	0	12.69	158.33	0.76
6	168.18	0	81.74	25.08	6.79	8.80	75.76	2.22
7	92.82	0	60.75	21.34	5.56	8.75	89.25	1.04
8	31.19	3.65	80.75	23.43	5.56	8.73	194.94	0.16
9	145.41	0	84.73	18.55	5.56	8.76	62.95	2.31
10	172.42	0	82.24	9.64	5.70	8.79	68.97	2.50
11	166.08	0	74.30	11.60	10.90	12.49	75.49	2.20
12	208.20	0	93.99	20.77	12.83	14.95	86.03	2.42
13	205.46	0	103.95	9.13	10.93	13.93	92.55	2.25
14	179.84	0	1.60	12.49	5.62	14.79	75.88	2.37
15	313.96	8.32	2.03	30.57	11.19	313.96	365.07	0.86
16	104.59	3.65	1.66	13.20	6.22	0	190.16	0.55
17	166.68	0	72.48	7.76	5.67	8.73	38.76	4.30
18	110.96	0	73.51	7.74	5.71	10.45	46.43	2.39
Min.	31.19	0.00	1.60	0.00	0.00	0.00	38.76	0.16
Max.	313.96	13.84	122.03	59.98	29.79	313.96	485.60	4.30
Mean	160.30	1.64	69.04	19.60	10.55	30.71	142.12	1.72
SD	68.65	3.75	35.79	14.48	8.83	71.77	116.33	1.08

*Concentration calculated indirectly through dividing the under curve area of heroin by the under the curve area of acetylcodeine and multiplying the result by the heroin concentration.

**Proportion of under curve area of heroin to the under the curve area of acetylcodeine (no unit).

## Discussion

The present study on the chemical combination of Iranian crack showed that this substance contains heroin, codeine, caffeine, morphine, thebaine, acetaminophen and a significant amount of acetylcodeine. None of the specimens showed stimulant substances such as cocaine, medicines and impurities such as sedatives (*e.g*. benzodiazepines and barbiturates), tricyclic antidepressants, aspirin or other synthetic opioids (*e.g*. tramadol and *buprenorphine*). The proportion of heroin to acetylcodeine was low approximately in half of the specimens, indicating the high concentration of acetylcodeine and higher toxicity in those specimens.

In line with the experiential findings of addiction treatment practitioners in that the clinical sings and also the overdose signs of Crack appear to be similar to heroin (7), our findings showed that the Iranian crack is heroin-based and hence different from those found in the West. Heroin and its related compounds were found in all our specimens. In the qualitative study by Kazemifar *et al*. all 22 but one specimens obtained from a city in central Iran contained heroin or its derivatives (4). The results of these studies show that heroin and heroin derivatives constitute the principal substance in the Iranian crack at this time, similar to other studies on heroin (8-10). As another finding, these compounds have higher concentration level than typical heroin. In addition, the specimens in our study and Kazemifar *et al*.'s revealed a wide degree of diversity in compounds in the Crack. Compounds such as caffeine and acetaminophen were found in our study while Kazemipour *et al*. found caffeine, dextromethorphan and chloroquine (4).

The impurities in the heroin preparation place are partially due to the alkaloid leftovers, such as morphine, codeine and thebaine and partly due to heroin byproducts such as monoacetyl morphine and acetylcodeine (11-14). Other compounds (such as acetaminophen and caffeine) originate from added impurities. It is common all over the world to adulterate heroin before selling it to users (11-16). Impurities can be classified into two groups: additives that increase the bulk, such as sugars (*e.g*. glucose, lactose, sucrose and mannitol) and ascorbic acid and substances such as caffeine, procaine, strychnine, quinine and acetaminophen that embitter heroin and conceal the sweetness of the buffing materials (17). In addition to adding bulk and a bitter taste, some of these substances, such as caffeine and acetaminophen, expedite heroin evaporation by heat and increase its effect by smoking (18).

In line with the findings of other studies on illegal drugs, we also found that our specimens were quite different from each other and they showed different proportions of the constituent compounds (17). Similar findings have also been shown in studies on street heroin (17). The amount of morphine, codeine and acetyl products and the amount of heroin and acetylcodeine and their ratio in the illegal specimens of heroin were used as basic criteria for comparing the specimens and their origin of preparation. It has been observed that theoretically the heroin-acetylcodeine ratio doubles through each stage of chemical conversion of opium to morphine to heroin. The heroin-acetylcodeine ratio in the opium specimens shows significant change from the origin of preparation which is indicative of alkaloid compounds in each area. These profiles allow the comparison of heroin specimens in different areas with illegal heroin specimens of unknown origin and also allow us to determine the geographical place where heroin was made (11, 19).

Although the chemical combination of Crack is very similar to that of the street heroin, it seems that this drug is not exactly concentrated heroin in terms of physical characteristics, usage route or chemical features. Unlike heroin, Crack is solid and inodorous. In addition, we assert that Iranian crack can be used by needle without the need for foil and its injection does not need heating and most users were ex-opium addicts, not heroin users (2). Another difference between Crack and street heroin is the proportion of acetylcodeine. Acetylcodeine (6-monoacetyle codeine) is an impurity which is formed in the street heroin due to poor baking and is a marker of street heroin (20). The street heroins all over the world contain 1% to 15% acetylcodeine (21) which may barely reach 45% (22). However, pharmaceutically produced heroin contains less than 0.1% acetylcodeine. In our study, most specimens of Crack showed a substantial amount of acetylcodeine. As Figure 2 shows, specimens can be classified into two groups by heroin-acetylcodeine ratio: ten specimens having a proportion of heroin 2-3 times (even four times for specimen #17) and eight other specimens with less heroin concentration. Although acetylcodeine in human body is metabolized into codeine and then into morphine, so it is less likely to be abused as a substance. Nevertheless, its overdose releases a great level of histamine in the blood and may lead to *anaphylactic shock*, convulsion and even death (23). Thus a higher concentration of acetylcodeine in the Crack (compared to street heroin) is indicative of higher toxicity.

It seems that the use of Crack is on the rise in Iran (2) as unlike heroin, opium and cannabis, it is odorless, easy to consume and needless to use many tools. In addition, the drug consumer can use the Crack in the bathroom or washroom in less than a minute or two without leaving any trace of odor in the space. However, the short-lived effect and instant tolerance causes faster withdrawal than heroin and follows higher dose and consequently shifting from sniffing to injection method (4, 7). Therefore, Iranian crack is more addictive and causes socio-economic fall for the individual. In addition, it has more devastating mental and physical harms than other forms of substances. Therefore, one may argue that although the Iranian crack is based on heroin and not on cocaine, its highly addictiveness nature and mental and psychological effects may ensue illegal financial activities or violent behaviors, as in crack cocaine (7, 24 and 25).

Because of added impurities to illegal drugs, the users are not well aware of their real power or components. Therefore, they are more subject to overdose or death, in particular when a new type of substance is released. Thus, identification of the chemical components of substances in any area, in particular for those new ones, can provide us both with valuable information about their origin and also for the treatment of their clinical signs. The present study is one of the few ones undertaking such an aim. However, this study is limited in several ways. First of all, the sample size was small (18 specimens) which is nevertheless justifiable considering the fact that it is an illegal drug and costly for studying samples. Secondly, all specimens were obtained from Tehran, which prevents the results to be generalized to the whole country. However, Kazemifar *et al*. produced similar results in a different city (4).

## Conclusion

The present study on the chemical combination of Iranian crack shows that it is a heroin-based substance and hence basically different from crack cocaine found in Western countries. However, the diversity of the components and their different amounts show that the production and distribution of this substance is diverse and may keep on changing into the future. Therefore, such studies at different time points, especially in relation to abnormal clinical signs, can provide relevant information about the clinical management of acute or chronic toxicity caused by these substances.

## References

[1] Ellenhorn MJ, Matthew J (1997). Ellenhorn’s Medical Toxicology.

[2] Farhoudian A, Rahimi Movaghar A, Mohammadi F (2008). “Crack”: The Most Notorious Drug: Results from a Qualitative Study in Iran. Proceeding of the 10th Annual meeting of International Society of Addiction Medicine.

[3] Narenjiha H, Rafiey H, Jahani MR, Assari S, Moharamzad Y, Roshanpazooh M (2009). Substance-dependent professional drivers in Iran: a descriptive study. Traffic Inj Prev.

[4] Kazemifar AM, Solhi H, Badakhshan D (2011). Crack in Iran: is it really cocaine? J. Addict Res. Ther..

[5] Souri E, Hatami A, Shabani Ravari N, Alvandifar F, Barazandeh Tehrani M (2013). Validating a Stability Indicating HPLC Method for Kinetic Study of Cetirizine Degradation in Acidic and Oxidative Conditions. Iran J. Pharm. Res..

[6] Odović J, Karljiković-Rajić K, Trbojević-Stanković J, Stojimirovic B, Vladimirov S (2012). The Lipophilicity Examination of Some ACE inhibitors and Hydrochlorothiazide on Cellulose in RP Thin-Layer Chromatography. Iran. J .Pharm. Res.

[7] Razani N, Mohraz M, Kheirandish P, Malekinejad M, Malekafzali H, Mokri A, McFarland W, Rutherford G (2007). HIV risk behavior among injection drug users in Tehran, Iran. Addiction.

[8] Klemenc S (2000). Noscapine as an adulterant in illicit heroin samples. Forensic Sci Int..

[9] Dams R, Benijts T, Lambert WE, Massart DL, De Leenheer AP (2001). Heroin impurity profiling: trends throughout a decade of experimenting. Forensic Sci Int..

[10] Gheorghe M, Bălălău D, Ilie M, Baconi DL, Ciobanu AM (2008). Component analysis of illicit heroin samples by GC-MS method. Farmacia.

[11] Van der Slooten EP, van der Helm HJ (1975). Analysis of heroin in relation to illicit drug traffic. Forensic Sci.

[12] O'Neil PJ, Baker PB, Gough TA (1984). Illicitly imported heroin products: some physical and chemical features indicative of their origin. J Forensic Sci..

[13] Huizer H (1983). Analytical studies on illicit heroin. I. The occurrence of O-3-monoacetylmorphine.. J. Forensic Sci..

[14] Huizer H (1983). Analytical studies on illicit heroin. II. Comparison of samples. J. Forensic Sci..

[15] Johnson DW, Gunn JW Jr (1972). Dangerous drugs: adulterants, diluents and deception in street samples. J. Forensic Sci..

[16] Eskes D, Brown JK (1975). Herion-caffeine-strychnine mixtures--where and why?. Bull. Narc.

[17] Kaa E, Bent K (1986). Impurities, adulterants and diluents of illicit heroin in Denmark (Jutland and Funen). Forensic Sci Int..

[18] United Nation Office on Drugs and Crimes (c) Afghanistan Identifies Cutting Agents for Heroin. http://www.unodc.org/unodc/en/frontpage/2009/June/afghanistan-identifies-cutting-agents-for-heroin.htm.

[19] Narayanaswami K (1985). Parameters for determining the origin of illicit heroin samples. Bull Narc..

[20] Phillips SG, Allen KR (2006). Acetylcodeine as a marker of illicit heroin abuse in oral fluid samples. J Anal. Toxicol..

[21] O’Neal CL, Poklis A (1998). The detection of acetylcodeine and 6-acetylmorphine in opiate positive urine. Forensic Sci Int..

[22] Soine WH (1986). Clandestine drug synthesis. Med Res. Rev..

[23] O'Neal CL, Poklis A, Lichtman AH (2001). Acetylcodeine, an impurity of illicitly manufactured heroin, elicits convulsions, antinociception, and locomotor stimulation in mice. Drug Alcohol Depend.

[24] Oliveira LG, Ponce Jde C, Nappo SA (2010). Crack cocaine use in Barcelona: a reason of worry. Subst Use Misuse.

[25] Vaughn MG, Fu Q, Perron BE, Bohnert AS, Howard MO (2010). Is crack cocaine use associated with greater violence than powdered cocaine use? Results from a national sample. AmJ. Drug Alcohol Abuse.

